# Improvement of critical current density of *RE*Ba_2_Cu_3_O_7-δ_ by increase in configurational entropy of mixing

**DOI:** 10.1098/rsos.211874

**Published:** 2022-03-30

**Authors:** Aichi Yamashita, Yuta Shukunami, Yoshikazu Mizuguchi

**Affiliations:** Department of Physics, Tokyo Metropolitan University, 1-1 Minami-Osawa, Hachioji, Tokyo 192-0397, Japan

**Keywords:** high-entropy, material design, cuprate superconductor

## Abstract

*RE*Ba_2_Cu_3_O_7-_*_δ_* (*RE*123, *RE*: rare earth) is one of the high-temperature superconductors with a transition temperature (*T*_c_) exceeding 90 K. Because of its high-*T*_c_ and large critical current density (*J*_c_) under magnetic fields, *RE*123 superconductors have been expected to play a key role in superconductivity application. To accelerate application researches on *RE*123-based devices, further improvements of *J*_c_ characteristics have been desired. In this study, we investigated the effects of high-entropy alloying at the *RE* site on the superconducting properties, through the measurements of local (intra-grain) *J*_c_ ($J_{c}^{\mathrm{local}}$) by a remanent magnetization method. We found that $J_{c}^{\mathrm{local}}$ shows a trend to be improved when four or five *RE* elements are mixed at the *RE* site, which results in high configurational entropy of mixing (Δ*S*_mix_). All samples exhibited an order of few MA cm^−2^ which is a criterion for practical application and the highest $J_{c}^{\mathrm{local}}$ resulted in a value of around 7.0 MA cm^−2^ at *T* = 2.0 K. Because high-entropy alloying can improve $J_{c}^{\mathrm{local}}$ of *RE*123 superconductors, our entropy-engineering strategy introduced here would be useful for the development of *RE*123 superconducting materials available under high magnetic fields.

## 1. Introduction

Since the discovery of high-transition temperature (high-*T*_c_) Cu-oxide superconductor [1], various kinds of Cu-oxide superconductors have been discovered [2–5]. Among them, *RE*Ba_2_Cu_3_O_7-_*_δ_* (*RE*123, *RE*: rare earth) in a thin-film form is a promising material for high-field superconductivity application because of its high-*T*_c_ exceeding 90 K and high critical current density (*J*_c_) under magnetic fields [6]. To improve *J*_c_ of *RE*123 films, nanoscale disorders such as nanoparticles, nanocomposite structure, defects etc. were introduced [7–10]. However, the current record of *J*_c_ of *RE*123 films are far from the limit expected for an ideal *RE*123 material [11]. To achieve higher *J*_c_ in *RE*123 materials, further development of the method for *J*_c_ engineering is needed. Having considered the structural and physical properties of various Cu-oxide superconductors, we find that the *RE*123 system is relatively difficult to use in superconductivity applications because it contains structural and compositional fluctuations. High-*T*_c_ and high-*J*_c_ superconductivity of *RE*123 is generally observed in the orthorhombic structure with the space group of *Pmmm* (#47) [12]. Decrease in oxygen amount in *RE*123 in the blocking layer results in a decrease in hole carriers and suppression of high performance of *RE*123. Recently, the improvement of *J*_c_ in *RE*123 film was achieved by over-doping of holes [13]. The improvement of *J*_c_ by chemical-composition tuning would be a desired progress because that can be applied together with the nanoscale fabrication techniques mentioned above. Here, we show another strategy to improve local (intra-grain) *J*_c_ ($J_{c}^{\mathrm{local}}$) by introducing high-entropy-alloy-type (HEA-type) *RE* site in *RE*123 superconductor.

HEA is an alloy containing five or more elements with a concentration range between 5 and 35 at% and hence has a high configurational entropy of mixing (Δ*S*_mix_), which is defined as Δ*S*_mix_ = −*R Σ_i_ c_i_* ln *c_i_*, where *c_i_* and *R* are compositional ratio and the gas constant, respectively [14,15]. Although the field of HEA had mostly focused on structural materials for the use under extreme conditions, various functionalities have been found in HEAs [15,16]. In 2014, superconductivity was observed in a HEA, Ti-Zr-Hf-Nb-Ta [17]. Although the expected pairing mechanisms of superconductivity for the HEA was a conventional type, the unique structural and compositional character were welcomed in the field of new superconducting materials. As reviewed in [18] and [19], many HEA superconductors were discovered after the first discovery by Koželj *et al*. [17]. Since 2018, we have developed HEA-type superconducting compounds, in which the HEA concept was applied to complicated compounds having two or more crystallographic sites [20]. Comparing the HEA effects for superconductors with various crystal structural dimensionality, we found that the disordering effects introduced by the HEA-type site in layered system (BiS_2_-based superconductor) [21] and quasi-two-dimensional system (tetragonal *Tr*Zr_2_, *Tr*: transition metals) [22,23] does not suppress its original *T*_c_ in pure phases. By contrast, in three-dimensional systems (NaCl-type metal tellurides [24,25] and A15 niobium-based compounds [26]), *T*_c_ of HEA-type phases was clearly lower than that for pure phases. Therefore, in a two-dimensional crystal structure, the introduction of HEA-type site does not negatively work on *T*_c_ of the superconductor. As a result, we previously reported the synthesis of HEA-type *RE*123 polycrystalline samples and reported that the increase in Δ*S*_mix_ does not suppress superconducting properties including *J*_c_ [27]. Let us remind that the technique of mixing *RE* elements has been used for the improvement of *J*_c_ in some *RE*123 materials [28]. To the best of our knowledge, however, superconducting properties including *J*_c_ for *RE*123 with remarkably high Δ*S*_mix_ at the *RE* site was examined in [27] for the first time. In this study, we expanded the study and synthesized various samples of *RE*123 using lighter *RE* elements including Dy, Ho, Yb and Lu. Here, we show that tuning Δ*S*_mix_ at the *RE* site could improve intra-grain *J*_c_ ($J_{c}^{\mathrm{local}}$) of *RE*123 superconductors.

## 2. Material and methods

Polycrystalline samples of *RE*Ba_2_Cu_3_O_7-_*_δ_* (*RE*: Y, La, Nd, Sm, Eu, Dy, Ho, Yb and Lu) were prepared by solid-state reaction and all samples were prepared in air, as described in [26]. Powders of Y_2_O_3_ (99.9%), Sm_2_O_3_ (99.9%), Eu_2_O_3_ (99.9%), Dy_2_O_3_ (99.9%), Ho_2_O_3_ (99.9%), Yb_2_O_3_ (99.9%), Lu_2_O_3_ (99.9%), BaCO_3_ (98%) and CuO (99.9%) were used for the synthesis. To obtain the best superconducting properties of each sample, two-step or three-step sintering was performed in air. Note that annealing in air at a low temperature around 350°C is performed because *T*_c_ of *RE*123 decreases with lower oxygen amount. Three-step sintering was performed for all samples except for labelled RE-2 and RE-3 (table 1 for the composition and electronic supplementary material, table S1 for the condition). We also confirmed that sample which showed enough high-*T*_c_ exhibited almost same superconducting properties after the three-step sintering The raw chemicals with a nominal compositional ratio of *RE* : Ba : Cu = 1 : 2 : 3 were well mixed and pelletized with a diameter of 1 cm. The first sintering condition was 930°C for 20 h, followed by furnace cooling. For the second sintering process, the sample was ground, mixed, pelletized in the same manner as the first one and heated at 930°C for 8 h and 350°C for 18 h, followed by furnace cooling. The third sintering was performed in three-step sintering for samples which showed low *T*_c_ and shielding volume fraction (SVF) after the second sintering. The condition of the third sintering was 930°C for 8 h, 350°C for 18 h and 175°C for 12 h, followed by furnace cooling.

**Table 1.** Compositional, structural and superconducting properties of *RE*Ba_2_Cu_3_O_7-_*_δ_* samples examined in this study.

label	*RE* site (EDX)	Δ*S*_mix_ (*RE*)	*a* (Å)	*b* (Å)	*OP*	*T*_c_ (K)
RE-1	Y	0	3.81375(10)	3.8804(2)	0.0173	92.9
RE-2	Y_0.57_Nd_0.43_	0.68*R*	3.85007(13)	3.9126(2)	0.0161	92.2
RE-3	Y_0.39_Sm_0.30_Eu_0.31_	1.09*R*	3.84149(10)	3.9038(2)	0.0161	93.2
RE-4	Y_0.32_Sm_0.22_Eu_0.26_Dy_0.20_	1.37*R*	3.8340(2)	3.89927(10)	0.0169	92.8
RE-5	Y_0.24_Sm_0.15_Eu_0.17_Dy_0.16_Ho_0.28_	1.58*R*	3.8290(2)	3.89593(8)	0.0173	93.0
RE-6	Y_0.16_Sm_0.14_Eu_0.15_Dy_0.14_Ho_0.14_Yb_0.27_	1.76*R*	3.8163(3)	3.8858(12)	0.0180	92.0
RE-7	Y_0.15_Sm_0.10_Eu_0.12_Dy_0.12_Ho_0.17_Yb_0.22_Lu_0.12_	1.91*R*	3.81933(14)	3.89045(10)	0.0185	91.4

Powder X-ray diffraction (XRD) patterns were collected on MiniFlex-600 (RIGAKU), equipped with a D/tex-Ultra high-resolution detector, with a Cu-K*α* radiation by a conventional *θ*−2*θ* method. Rietveld refinement was performed using RIETAN-FP [29]. Crystal structure images were drawn using VESTA [30]. The actual composition of the synthesized disc-shaped polycrystalline samples was investigated by energy-dispersive X-ray spectroscopy (EDX) on a scanning electron microscope (SEM), TM-3030 (Hitachi), with Swift-ED (Oxford). The compositions were estimated by averaging the EDX analysis result from four points on the surface of the examined samples. The obtained compositions are shown in electronic supplementary material, figure S1 with SEM images.

The superconducting properties were investigated using a superconducting quantum interference device magnetometer on MPMS-3 (Quantum Design). For the precise measurement, the disc-shaped samples were cut into the cube-shaped form with a typical size of 0.18 × 0.17 × 0.20 mm. The temperature dependence of magnetic susceptibility (4π*χ*) was measured after both zero-field cooling and field cooling with an applied field of approximately 10 Oe. The temperature dependences of susceptibility for all the samples are shown in electronic supplementary material, figure S2, and the estimated *T*_c_ is listed in table 1. All samples exhibited high-*T*_c_ and large SVF, indicating that the samples were oxygenated enough. To estimate $J_{c}^{\mathrm{global}}$ of cube-shaped samples, magnetization-magnetic field (*M*-*H*) loops were measured. From the obtained *M*-*H* loops, $J_{c}^{\mathrm{global}}$ was estimated using the Bean's model [31]: *J*_c_ = 20Δ*M*/*B*(1-*B*/3*A*) (A cm^−2^), where *A* and *B* are lengths determined by sample shape, and Δ*M* is obtained from the width of the *M*-*H* curve. Typical results on $J_{c}^{\mathrm{global}}$ are plotted as a function of field in the electronic supplementary material, figure S3. To estimate $J_{c}^{\mathrm{local}}$, remanent magnetization (*m*_R_) was measured. Remanent magnetization is defined to be a residual magnetization after the applied magnetic field is turned off [32]. When the field is applied to the sample, two full penetration fields at *H*_p1_ and *H*_p2_ appear at lower field and higher field. When the sample is partially penetrated by the lower field, the contribution to remanent magnetization or the total trapped flux by the pinning centres is only located in the thin penetrated layer. The sample is first fully penetrated with an increasing field at *H*_p1_. As the applied field further raises, more flux lines get over the pinning barrier and enter the sample. The stronger the pinning strength or the higher the applied field, the more flux lines trapped. The trapped flux lines contribute highly to the remanent magnetization, which has a large value compared with the first full penetration field *H*_p1_. Therefore, the remanent magnetization sharply increases with increasing applied field until the second full penetration field *H*_p2_. The derivative of *m*_R_ with maximum magnetic field (*H*_m_) is given by the following equation with respect to the three different *H*_m_ conditions [33], $dm_{R}/\mathrm{dlog}H_{m}=(3\pi^{2}r^{3}H_{m}/4H_{\;p2})(1-3H_{m}/2H_{\;p2}+7H_{m}^{2}/12H_{p2}^{2})$: 0 < *H*_m_ ≤ *H*_p2_, $\left(\pi^{2}r^{3}H_{\;p2}/8)(4/H_{\;p2}-6H_{m}/H_{p2}^{2}+3H_{m}^{2}/H_{p2}^{3}-H_{m}^{3}/2H_{p2}^{4}\right)$: *H*_p2_ < *H*_m_ < 2*H*_p2_, and 0: *H*_m_ > 2*H*_p_. In the equation, d*m*_R_/dlog*H*_m_ exhibits a peak at $H_{m}=H_{\mathrm{peak}}=(6-2\sqrt{2})Hp2/7$. Therefore, $J_{c}^{\mathrm{local}}$ can be calculated from $H_{p2}=J_{c}^{\mathrm{local}}r$, where *r* is average grain size. The *H*_m_ dependence of d*m*_R_/dlog*H*_m_ was measured using a sequence for remanent magnetization measurements. First of all, *H*_m_ was applied to the sample, and the magnetic field was set to zero, followed by magnetization measurement, which gives *m*_R_ (*H*_m_). The same measurements were performed at different maximum fields and temperatures. From the *H*_m_ dependence of d*m*_R_/dlog*H*_m_, *H*_p2_ was estimated from the peak position *H*_peak_. The cube-shaped samples were crushed into fine powders by grinding using mortar and pestle. The powders close to 20 µm were selected by micro sieves and used for remanent magnetization measurements. The diameter of powders was estimated using ImageJ [34].

## 3. Results and discussion

**Figure 1.**
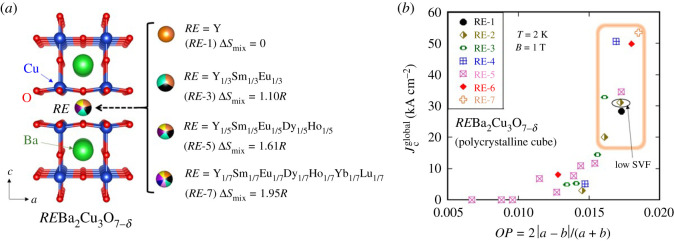
Structural and in-field $J_{c}^{\mathrm{global}}$ characteristics of polycrystalline *RE*Ba_2_Cu_3_O_7-_*_δ_*. (*a*) Schematic images of crystal structure of *RE*Ba_2_Cu_3_O_7-_*_δ_* and high-entropy alloying at the *RE* site. The Δ*S*_mix_ was calculated according to the nominal values of *RE* concentration. (*b*) Magnetic $J_{c}^{\mathrm{global}}$ (*T* = 2 K, *B* = 1 T) as a function of orthorhombicity parameter (*OP* = 2 |*a* − *b*|/(*a* + *b*)). In this study, we examined $J_{c}^{\mathrm{local}}$ for samples surrounded by an orange square in the (*b*). Because of low SVF estimated from temperature dependence of susceptibility, one RE-2 sample containing Y and Sm was excluded in this study. Some of the $J_{c}^{\mathrm{global}}$ data have already been published in [27].

Figure 1*a* shows a schematic image of the crystal structure of HEA-type *RE*123 and the concept of HEA-type site at the *RE* site. With increasing number of *RE* in the site, Δ*S*_mix_ (ideal value) increases and exceeds 1.5*R* when five *RE* ions are mixed. Therefore, *RE*123 samples with more than four *RE* ions are expected to be HEA-type *RE*123. In figure 1*b*, inter-grain global *J*_c_ ($J_{c}^{\mathrm{global}}$) estimated from the *M*-*H* loops is plotted as a function of orthorhombicity parameter (*OP*): *OP* = 2|*a* − *b*|/(*a* + *b*). Here, *OP* was used to discuss the superconducting properties because, as mentioned earlier, orthorhombicity is a key structural parameter to achieve higher superconducting characteristics in the *RE*123 system [12,27]. Since *OP* is a good scale for estimating oxygen deficiency in the *RE*123 system, the use of *OP* in plotting superconducting properties is useful to extract the effects of Δ*S*_mix_ at the *RE* site of *RE*Ba_2_Cu_3_O_7-_*_δ_* on the *J*_c_ characteristics in the system. According to the data in figure 1*b*, we found that higher $J_{c}^{\mathrm{global}}s$ are achieved for samples containing four, six and seven *RE* elements. The results motivated us to study $J_{c}^{\mathrm{local}}$ of *RE*123 samples with different Δ*S*_mix_ to clarify the effect of high configurational entropy of mixing to *J*_c_ characteristics. Then, seven samples having different Δ*S*_mix_ and almost comparable *OP* were selected for this study (highlighted with a square in figure 1*b*). As well, high enough *T*_c_ and bulk nature of superconductivity of the sample was confirmed through *M*-*T* measurements as displayed in the electronic supplementary material, figure S1 after oxygenation. These indicating that the samples were oxygenated enough, and *OP* are high enough and not a critical parameter to consider for $J_{c}^{\mathrm{global}}$ and $J_{c}^{\mathrm{local}}$. According to the number of *RE* elements, examined samples are labelled RE-1–RE-7 (table 1).

**Figure 2.**
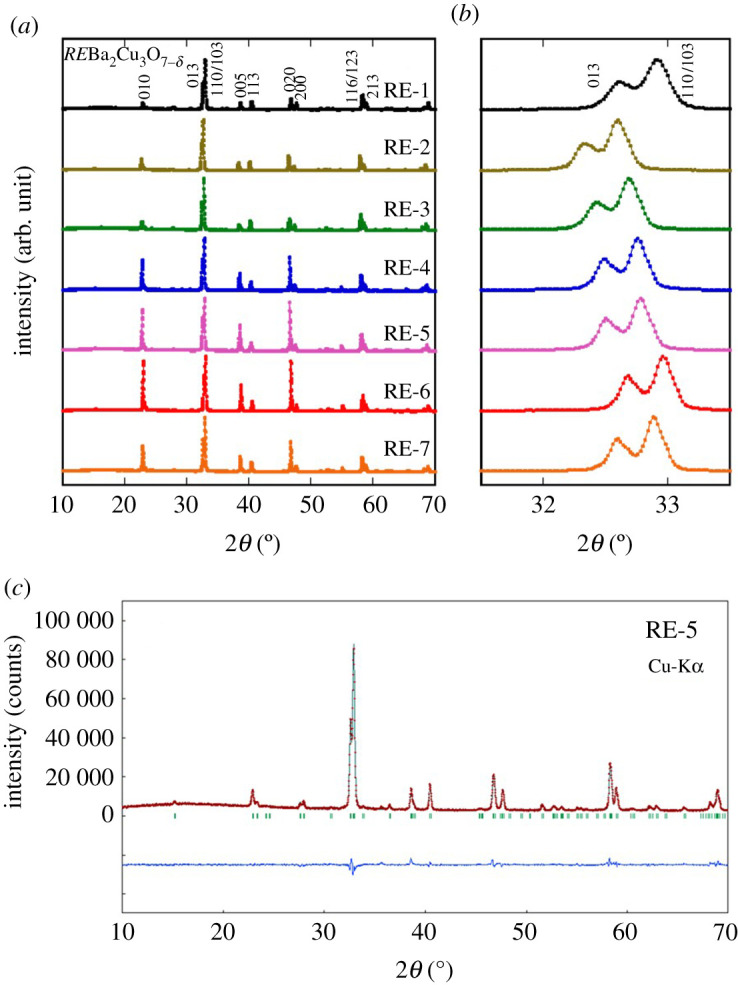
XRD patterns of *RE*Ba_2_Cu_3_O_7-_*_δ_* samples. (*a*) Powder XRD patterns of all the examined samples, RE-1–RE-7. (*b*) Zoomed plots near the (013) peak for RE-1–RE-7. (*c*) Rietveld refinement result for RE-5.

**Figure 3.**
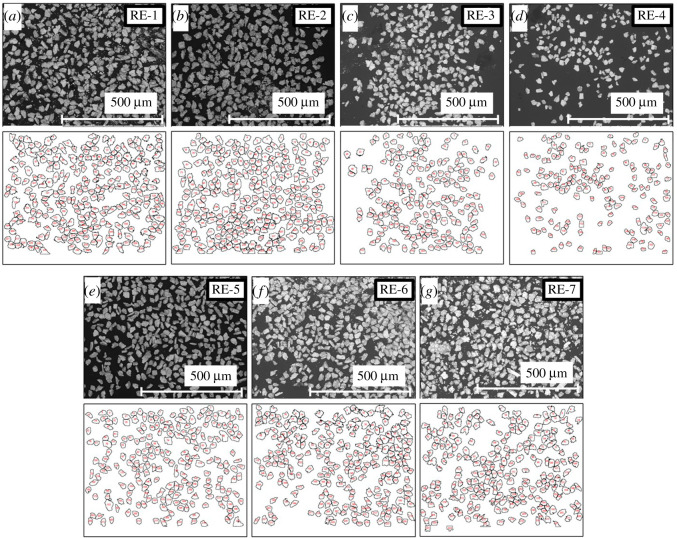
Powder characterization. (*a*–*g*) SEM images of *RE*Ba_2_Cu_3_O_7-_*_δ_* powders (upper panels) and diameter analyses (lower panels) for all the samples, RE-1–RE-7. SEM images were analysed using ImageJ software in [33].

Through EDX analyses for cube-shaped samples (see electronic supplementary material, figure S1), we confirmed that the actual composition of the examined samples is comparable to the nominal value, as summarized in table 1. Figure 2*a* shows powder XRD patterns for all the samples (RE-1–RE-7). All the peaks could be indexed with the orthorhombic *RE*123 model with a space group of *Pmmm* (#47). As displayed in figure 2*b*, no peak broadening was observed among seven samples, indicating that the increase in Δ*S*_mix_ does not affect crystallinity of the polycrystalline *RE*123 samples. The major peaks shifted according to the average ionic radius at the *RE* site. The shift of peaks from RE-2 to RE-7 is originated from the systematic increase of *RE* element with smaller ionic radius such as Sm, Eu, Dy, Ho, Yb and Lu. As mentioned earlier, however, in the *RE*123 system, *OP* is the essential parameter for superconducting properties rather than lattice constants. We, therefore, estimated lattice constants, *a* and *b*, using the Rietveld refinements. The typical refinement result with a reliability factor, *R*_wp_ = 5.8%, for RE-5 is shown in figure 2*c*, which shows that the orthorhombic model can nicely reproduce the XRD patterns even for a HEA-type sample with five different *RE*. The estimated lattice constants and the calculated *OP* are summarized in the electronic supplementary material, table S1. To perform remanent magnetization (*m*_R_) measurements, powders with similar diameter are prepared and observed by SEM. As shown in figure 3, the diameter of the powders was almost homogeneous, and the estimation of the average diameter was successful using ImageJ software. The estimated average diameter of powders for RE-1–RE-7 is summarized in table 2.

**Table 2.** Average area and diameter of the *RE*Ba_2_Cu_3_O_7-_*_δ_* powders used for the estimation of $J_{c}^{\mathrm{local}}$.

label	average area (μm^2^)	average diameter (μm)
RE-1	472.23	21.7
RE-2	508.567	22.6
RE-3	420.793	20.5
RE-4	321.049	17.9
RE-5	427.58	20.7
RE-6	438.918	21.0
RE-7	437.66	20.9

**Figure 4.**
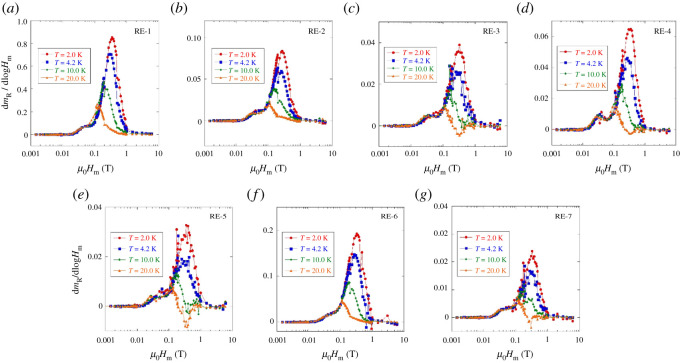
Results of remanent magnetization measurements. (*a*–*g*) Maximum magnetic field (*μ*_0_*H*_m_) dependences of the *H*_m_ derivative of remanent magnetization (*m*_R_), in a form of d*m*_R_/dlog*H*_m_, for RE-1–RE-7.

**Figure 5.**
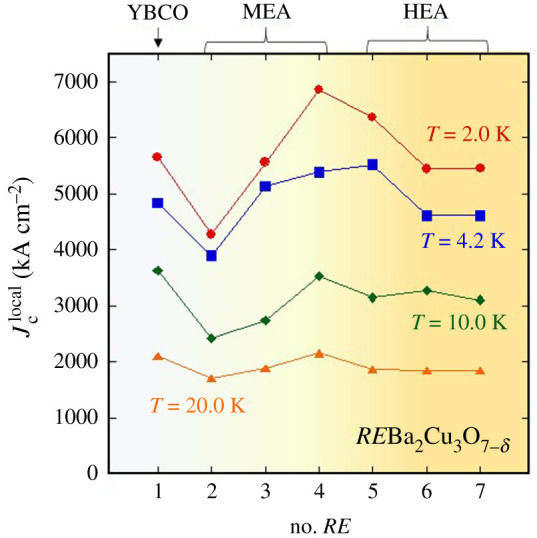
Estimated $J_{c}^{\mathrm{local}}$ as a function of number of *RE* elements.

Figure 4 shows the results of the *m*_R_ measurements at *T* = 2.0, 4.2, 10.0, 20.0 K plotted in a form of d*m*_R_/dlog*H*_m_ as a function of maximum applied field (*H*_m_). Basically, we observed two peaks in the plots; the first peak observed at lower fields is originating from $J_{c}^{\mathrm{global}}$. Since powder samples were used for the *m*_R_ measurements, $J_{c}^{\mathrm{global}}$ is reasonably quite low. The second peak at higher fields is originating from $J_{c}^{\mathrm{local}}$. From the peak position (*H*_peak_), $J_{c}^{\mathrm{local}}$ was calculated using the equations $H_{\mathrm{peak}}=(6-2\sqrt{2})\;H_{p2}/7$ and $H_{p2}=J_{c}^{\mathrm{local}}r$, where *r* is average grain size, as described in Material and methods. The estimated $J_{c}^{\mathrm{local}}$ at *T* = 2.0, 4.2, 10.0, 20.0 K for RE-1–RE-7 is plotted in figure 5 as a function of number of *RE* elements. At 20.0 K, there is no clear difference in $J_{c}^{\mathrm{local}}$ among the seven samples except for slightly higher values for RE-1 and RE-4. At lower temperatures, the difference in $J_{c}^{\mathrm{local}}$ becomes remarkable. At 4.2 K, $J_{c}^{\mathrm{local}}$ of RE-3, RE-4 and RE-5 is higher than that of RE-1. Furthermore, at 2.0 K, $J_{c}^{\mathrm{local}}$ of RE-4 and RE-5 is clearly higher than that of RE-1, RE-2 and RE-3. Our results on $J_{c}^{\mathrm{local}}$ measured for the polycrystalline *RE*123 powders suggest that optimization of Δ*S*_mix_ at the *RE* site can improve $J_{c}^{\mathrm{local}}$ of *RE*123 materials at low temperatures.

Having compared the *OP* parameter for RE-1, RE-4 and RE-5, we found that *OP* for those samples is almost the same. Therefore, the oxygen amount would be comparable in those samples. Therefore, the improvement of $J_{c}^{\mathrm{local}}$ by the increase in Δ*S*_mix_ observed between RE-1, RE-4 and RE-5 would be originating from local structural modification by the disordered *RE* site and chemical bonds near the *RE* site. In BiS_2_-based *RE*(O,F)BiS_2_ superconductors, local structure modification in the conducting BiS_2_ layers by the increase in Δ*S*_mix_ was observed [35]. Therefore, we expect that structural modulation was generated in the CuO_2_ planes by the introduction of middle- or high-entropy-alloy-type *RE* site. The highly disordered *RE* site and the bonds would modify electronic states and microscopic characteristics of superconductivity in the samples. The lower $J_{c}^{\mathrm{local}}$ observed for RE-6 and RE-7 than that for RE-4 or RE-5 may be caused by huge disordered states. Although we have no clear scenario of the enhancement of pinning properties by the increase in Δ*S*_mix_, our current results encourage further studies on the relationship between configurational entropy of mixing and critical current properties in the *RE*123 materials. Synthesis of single crystals or thin films of HEA-type *RE*Ba_2_Cu_3_O_7-_*_δ_* and investigation on local structure modulations and microscopic characteristics of superconductivity are needed to clarify the HEA effects in *RE*123.

## 4. Conclusion

Here, we reported the improvement of $J_{c}^{\mathrm{local}}$ in *RE*Ba_2_Cu_3_O_7-*d*_ by the increase in Δ*S*_mix_ at the *RE* site. Polycrystalline *RE*123 samples with different Δ*S*_mix_ were synthesized by solid-state reaction. Through characterization of structural (lattice constants and *OP*), compositional and superconducting properties (*T*_c_ and shielding fraction), seven samples labelled RE-1–RE-7 were chosen, and remanent magnetization measurements were performed on those samples. At higher temperatures (*T* = 20.0 K), clear difference in $J_{c}^{\mathrm{local}}$ was not observed. At lower temperatures (*T* = 2.0 and 4.2 K), higher $J_{c}^{\mathrm{local}}$ was observed for RE-4 and RE-5 with a higher Δ*S*_mix_ as compared with that for RE-1, RE-2, and RE-3 with zero or low entropy of mixing at the *RE* site. Although the results of the current work showed the merit of high-entropy alloying at lower temperatures only, there should be optimal conditions on constituent *RE* element, mixing ratio and Δ*S*_mix_, which will achieve higher $J_{c}^{\mathrm{local}}$ at higher temperatures as well. If the trial was achieved, the HEA concept can be applied to all *RE*123 practical materials to additionally improve their critical current properties.

## Data Availability

The datasets supporting this article have been uploaded as part of the electronic supplementary material [36].
